# Nursing Institutions and Metropolitan Hospitals

**Published:** 1887-02-19

**Authors:** E. G. Manson

**Affiliations:** Matron of St. Bartholomew's Hospital


### A Noble Profession: Nursing the Sick.

### NURSING INSTITUTIONS AND METROPOLITAN HOSPITALS.

By Miss E. G. Manson,
Matron of St. Bartholomew's Hospital.

*Is it desirable for metropolitan hospitals to possess nursing institutions and to supply nurses for private cases, outside the hospital walls?*

Until recent times, the great idea with regard to the sphere of the hospital seems to have been, not how far its useful influence and its work could be extended, but to what narrow limits it could be reduced. A hospital was, and is still, in the eyes of a great part of the public, simply an asylum for the sick poor, and it has, in their opinion, no other duties, no other responsibilities, than those of tending and doctoring them. There its function begins and there it ends. Others, on the contrary, look upon hospitals chiefly as institutions to which are attached medical schools, and which are intended more particularly for the benefit of medical science. It is there the doubtful problem can be worked out; with the aid of hospital comforts and appliances the new operation can be tried; the effect of the newest drug can be observed. Still more lately there has arisen yet another party, to whom the hospital is chiefly an object of interest as a training school for nurses. The more modern and enlightened view of a hospital embraces a proper appreciation of all these three points. By allowing none of these three important duties to be lost sight of, it has been clearly proved how each can be of the greatest help to the other, and by paying due regard to the legitimate demands of each department, the whole of the hospital is equally benefited.

A hospital to which is attached a medical school commands the highest members of the profession, who, while they assist in training future generations of doctors, give the sick poor the benefit of the best medical advice. When the nursing of a hospital is taught on the most thorough principle, the women who are attracted to it are of a class whose intelligence and education make them capable of being trained to a very high standard of proficiency; and it is when the organisation, the medical treatment, and the nursing are all of the very best quality, that the patient receives the greatest benefit from the hospital. Therefore, to place the patient in every way in the most favourable position for combating his disease, all hospitals should be conducted with a due regard for the proper development of all these departments. Few will deny that in good hospitals this is done, to the very best of our present knowledge, and the poor reap the fullest benefit in every way from hospitals.

But these are supported by the wealthy, who are certainly entitled to reap equal benefit from them. They obtain by payment, it is true, the advantage of the physicians' or surgeons' skill, learned by clinical observation in hospital wards, and in the same manner they should be able to obtain skilled nursing, perfected by daily practice in the wards, or the benefit of hospital appliances and accommodation modified to suit their requirements. There is no doubt that they have a right to these privileges; and the question as to how the sick rich can be best provided with really good nursing, without robbing the sick poor, is the one that is at present under consideration.

The want of hospitals and sick-nurses for the rich, as well as the poor, is one so marked, that it has had to be met in one way or another, and this is principally done at present by private nursing-homes, in the hands of private individuals, who farm out nurses for their own profit; a system that contains much that is obviously evil and unjust, both to the public and the nurses employed. At some of these homes women are engaged who have no previous experience of nursing. These are sent for six months' or a year's promiscuous training at some hospital, and then advertised as "hospital-trained nurses." Most institutions, however, draw their supply from among those who, for some reason or other, have left hospital work; and though the woman is usually required to bind herself for a certain number of years, those who make any stipulation as to the length of time she must have been trained, are few and far between.

As hospital authorities generally do their best to retain the services of their most competent nurses, and as the fact of belonging to a large, well-known hospital carries with it a certain amount of prestige, and the comforts of a well-disciplined organisation are sufficient to induce the better class of nurses to remain, in spite of the harder work and greater restrictions, it is only now and again that a highly competent nurse enters a private institution.

The nurses in a private institution being thus drawn indiscriminately from various hospitals and training schools, the manager of the establishment has to gain his very limited knowledge of their capabilities from testimonials and the nurse's own account of herself. Now, nurses are instruments that should be thoroughly known and tested by those who use them. In a well-organised hospital, the various faults or good qualities of any particular nurse are known to the authorities. Such and such a woman is good at medical work, careful and kind and observant, but wanting in that smartness which is indispensable in a good surgical nurse, a surgeon's assistant. Again, another is peculiarly skilful in the management of delirious patients—of fever cases; another possesses a large share of that boundless patience and forbearance necessary in nursing sick children, or that kind of firmness so essential in hysteria. But very few are equally skilful in all branches of their profession. It requires a large amount of discrimination and accurate knowledge of character to give to each nurse the post for which she is more particularly fitted; and in a hospital a great deal of care and tact are exercised in choosing suitable nurses for special cases. In most institutions little of that discrimination and tact can be, or at all events are used. An arrangement by which the authorities responsible for the nursing have no means of observing and judging the nurses' work, is necessarily faulty, and has given rise to many evils, which have called forth complaints both from the physicians and the public who have employed the trained nurses provided by many private institutions. In response to these complaints, and to supply the generally admitted want for trained certificated nurses, for whose proficiency competent authorities were able to vouch, the Westminster Hospital started its home for trained nurses, for private cases in connection with the hospital, and provided nurses trained in its own wards. So satisfactory were the results, and so generally appreciated was the undertaking, that one by one many of the other metropolitan hospitals followed suit, till even the most conservative took up the innovation with success.

The physicians and surgeons attached to the various hospitals were glad to avail themselves of women who had been trained to carry out their treatment in the manner best pleasing to them in their own wards, and the appreciation of the public was abundantly proved by the demand made on metropolitan hospitals for their skilled nurses. Nor is this all. Many hospitals have started beds or wards where, by payment, the middle classes can reap the benefit of hospital treatment and advice with a certain amount of that privacy, the loss of which often forms the one and only objection to a patient's entering the hospital. Many operations which can only be performed at an enormous expense and with great difficulty at the patient's own home, can be here carried out at a comparatively small cost and without difficulty. This arrangement is, however, still in its infancy, and it has only been adopted in a few hospitals, and the system is still very generally faulty and incomplete.

The question as to how best the sick rich can be benefited to the fullest extent by skilled hospital nursing and hospital treatment is one, therefore, the solution of which has been attempted in very different ways by different hospitals. There is no uniformity in their rules and arrangements. I shall make a few remarks as to what I consider the best way of organising a Private Nursing Institution attached to a large hospital, and the best system upon which to train nurses for the same.

The choice of women for private nursing is of the utmost importance, and hospital authorities should be very careful in their selection. Many who have the necessary qualities to make excellent ward-nurses are, from some fault in their appearance, address, or manner, unsuitable for private nursing. The nurse who is intended for private work should receive at least two years' training at the expense of the hospital to which she is attached, such training to be arranged on a system that will enable her to gain the greatest possible insight into the details of her profession during that time. She should spend the first twelve months of her training in the general, medical, and surgical wards of the hospital; care being taken to avoid as much as possible moving her oftener than once in three months; while her second year should be devoted to special branches, such as obstetrics, midwifery, ophthalmic cases, massage, and electricity. Having at its completion given satisfaction to the authorities as to her suitability in character and manners, as well as in her nursing qualifications, she should be taken entirely off the hospital books, and transferred to those of the attached nursing institution.

The system by which nurses for private cases are taken as required from the hospital wards and transferred back again as soon as they return, is most pernicious, and is manifestly unfair to the patients, as well as to the sister in charge and her subordinate nurses. It is destructive to the discipline and organisation of the wards, for the constant change interferes considerably with the routine of work and the regular carrying out of prescribed treatment. At all hospitals to which training schools are attached this evil has to be taken into consideration; but it should be the superintendent's care to *minimise* it to the utmost, and certainly not to add thereto by placing nurses in the wards who can never be counted upon with any certainty, and are liable to be removed at any moment, often when they are most wanted; but to prevent the nurse becoming rusty, she should from time to time, at such intervals as the Superintendent thinks fit, be transferred to the hospital, and stationed in a ward for a period of not less than three months. This would enable her to see any new dressing or instruments applied, any new treatment carried out, and to perfect herself anew in those details that are best practised in the routine of a hospital ward.

When not in charge of a case, the private nurses should live at a Home, separate from that of the ward-nurses, and under the charge of a separate Home sister. Their comfort should in every way be well provided for, so that their leisure time should be really a rest to them. They should have a liberal salary, and outdoor and indoor uniform, which they should be strictly enjoined always to wear when on duty. It is a perpetual reminder to them of the particular character under which they enter a house. It gives them a greater sense of their own responsibility, and more authority with their patient. It was only the other day that a lady who had been employing a nurse from a private institution, who had attended capless at an operation, said to me how much more confidence she should have felt in her nurse had she appeared in uniform and cap; for her experience of women (which was large) had led her to the conclusion that they always lived up to their outward appearance, and she felt convinced the nurse would have felt her responsibility and her position more had she worn her badge of office. Uniform should, both on this account and on hygienic grounds, always be worn by a nurse; washing material being supplied for sick-room wear, and a dark, woollen gown for travelling and outdoor exercise.

Certain rules and regulations should be drawn up, both for the guidance of the nurse and for her employers, a copy of which should always be sent to any house at which a nurse is employed. Such rules should include a provision for at least seven hours' uninterrupted sleep (when possible) for the nurse, and a sufficient supply of food. The public have often curious notions as to the amount of strain and fatigue that can be borne without rest or proper nourishment by a "trained nurse"; for while the patient should be, to a good and conscientious nurse, of paramount importance, her employers should never forget that the illness that is to them the one important event is to the nurse only one item in the routine of her life's work, and it is neither fair nor just to expect her to wear herself out and render herself unfit for her duties at her next post, by a selfish disregard of her comfort and health. I have heard people make bitter complaints of an otherwise excellent nurse, because "when we were all so busy, she would insist on going to bed for so many hours, and always wanted something hot for dinner."

Hospitals should provide none but good nurses; but as all cheap wares are nasty, while all good wares command and deserve a good price, they should not try to make too much money out of their nurses, but behave liberally towards them and protect their health and interests when on duty. It would be well if all those who employed nurses should afterwards forward to the Superintendent of the Home a report of the manner in which the nurse discharged her duties while with them. Any complaints that either the friends or the medical man had to make about her should also be addressed to the Superintendent.

The whole sphere of a hospital's usefulness is not complete without a private Home, where paying patients can be received; such Home or Hospital to be under the management of the Hospital Committee, and nursed by their own trained nurses: though it should not be restricted to the patients of members of the hospital staff, it would be most probably used by them or former students, and would be, properly managed and arranged, a very great boon to the public. Time, however, will not allow me to enter further into that topic.

The main point for which I am contending is briefly this—that private Nursing Homes are infinitely better managed, and infinitely more useful to the public, when attached to large, well-arranged hospitals and training schools, than when in the hands of private individuals. The nurses are more carefully selected, better supervised, and better cared for. Constantly living, when off duty, among surroundings where nursing is highly considered and holds an established position, raises the nurse's self-respect and her consideration for the dignity of her profession.

